# Dopamine and Noradrenaline in the Brain; Overlapping or Dissociate Functions?

**DOI:** 10.3389/fnmol.2019.00334

**Published:** 2020-01-21

**Authors:** Yadollah Ranjbar-Slamloo, Zeinab Fazlali

**Affiliations:** ^1^Eccles Institute of Neuroscience, The John Curtin School of Medical Research, The Australian National University, Canberra, ACT, Australia; ^2^Department of Biomedical Engineering, Columbia University, New York, NY, United States

**Keywords:** dopamine, noradrenaline, ventral tegmental area, Locus Coeruleus, co-transmission, neuromodulator, signaling

## Abstract

Dopamine and noradrenaline are crucial neuromodulators controlling brain states, vigilance, action, reward, learning, and memory processes. Ventral tegmental area (VTA) and Locus Coeruleus (LC) are canonically described as the main sources of dopamine (DA) and noradrenaline (NA) with dissociate functions. A comparison of diverse studies shows that these neuromodulators largely overlap in multiple domains such as shared biosynthetic pathway and co-release from the LC terminals, convergent innervations, non-specificity of receptors and transporters, and shared intracellular signaling pathways. DA–NA interactions are mainly studied in prefrontal cortex and hippocampus, yet it can be extended to the whole brain given the diversity of catecholamine innervations. LC can simultaneously broadcast both dopamine and noradrenaline across the brain. Here, we briefly review the molecular, cellular, and physiological overlaps between DA and NA systems and point to their functional implications. We suggest that DA and NA may function in parallel to facilitate learning and maintain the states required for normal cognitive processes. Various signaling modules of NA and DA have been targeted for developing of therapeutics. Understanding overlaps of the two systems is crucial for more effective interventions in a range of neuropsychiatric conditions.

## Introduction

Central nervous system produces diverse neurochemicals which bind to specific receptors coupled to the intricate intracellular signaling pathways. Synthesis and release of a primary neurotransmitter with a simplified action (e.g., excitatory or inhibitory) has been a ground for classification of neurons and synapses in the CNS. As knowledge of neurotransmission rapidly grew in the past few decades, it became soon clear that the actions of neurotransmitters are complex and some neurons produce and release two or more chemicals as fast neurotransmitters, neuromodulators, or neuropeptides (Vaaga et al., 2014). This often confounded the integration of connectivity knowledge with neurophysiology. Moreover, a single neurochemical can bind to different receptors and the receptor expression at the level of individual neurons are highly variable. For example, numerous metabotropic receptors have been identified for glutamate alone, with diverse distribution and function (Reiner and Levitz, 2018). Unlike glutamate and GABA which are known for their binding to the fast-acting ionotropic receptors, others predominantly activate metabotropic receptors, hence they are commonly called neuromodulators. These include monoamines such as noradrenaline (NA, also called norepinephrine), adrenaline (also called epinephrine), dopamine (DA), serotonin, and histamine. Monoamines are produced by small populations of neurons located in specific brain nuclei. These neurons project to widespread brain regions with numerous ramifications to broadcast specific signals about external stimuli and internal states.

**FIGURE 1:**
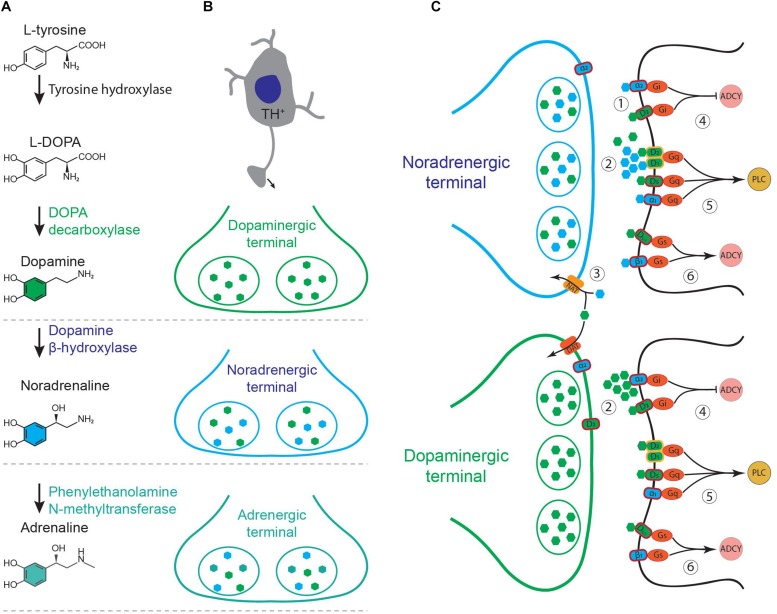
**(A)** Biosynthesis of catecholamines from L-tyrosine. **(B)** Schematic of a tyrosine hydroxylase positive (TH^+^) neuron and its axonal terminals containing different combination of catecholamines, depending on the type of the neuron. **(C)** Noradrenergic and dopaminergic terminals. (1) Co-release of dopamine (green) and noradrenaline (blue) and their binding to specific receptors on target neurons. (2) Non-specific binding at high concentrations. (3) Non-specific transporter function. NAT, noradrenaline transporter; DAT, dopamine transporter. (4–6) Intracellular pathways shared between noradrenergic and dopaminergic receptors. ADCY, adenylyl cyclase; PLC, phospholipase C.

The effect of neuromodulators on the target neurons is often mediated by G-protein coupled receptors. Multiple receptors may share a specific signaling pathway. Specificity of a neuromodulator then should depend on the source of release, concentration, efficacy, and the rate of depletion or reuptake from the extracellular space. Although the main neuromodulators, such as acetylcholine, dopamine, noradrenaline, and serotonin have a key role in controlling the brain states (Brown et al., 2012) and computations (Dayan, 2012), their interactions are yet mysterious. Dopamine and noradrenaline in particular, appear to significantly cross-talk in cortex and hippocampus (Devoto et al., 2006). Dopamine producing neurons are located in the midbrain nuclei; mainly ventral tegmental area (VTA) and substantia nigra pars compacta (Poulin et al., 2018). Noradrenergic nuclei are located in pons and medulla. Among these nuclei, LC contains the major proportion of the noradrenergic cells and targets multiple regions of the brain (Robertson et al., 2013). Although DA and NA have been studied as two separate systems, they overlap in multiple domains such as; shared biosynthetic pathway, co-release from noradrenergic terminals, innervation of similar area, non-specific receptor, transporter affinity, and shared intracellular signaling pathways (Figure 1). What are the functional consequences of these overlaps? In the following paragraphs, we expand the major domains of overlap and their neurophysiological and behavioral implications of such overlaps.

## Volume Transmission, Receptor Signalling, and Transporter Functions

Neuromodulators such as DA, NA, and acetylcholine can diffuse far from the release site and activate receptors in a considerable distance from the terminal (Agnati et al., 1995; Fuxe et al., 2010). This process, which is known as volume transmission, can activate receptors as far as 8 μm from the release site, in case of striatal dopamine (Sulzer et al., 2016). A long-range diffusion is also possible due to the circulation of the cerebrospinal fluid (CSF) along the peri-vascular space (Taber and Hurley, 2014). Peri-vascular CSF currents may supply neuromodulators far from their site of release, to activate receptors in area lacking direct projections.

Noradrenaline acts on three main G-protein coupled receptors, known as β-, α-1, and α-2 adrenoceptors (Figure 1C). These receptors have complex effects on neuronal excitability and synaptic transmission, depending on their site of action and concentration of NA (Berridge and Waterhouse, 2003; Arnsten et al., 2012; Waterhouse and Navarra, 2019). β-adrenoceptors are coupled to G_s_ which enhances cAMP signaling while α_2_-adrenoceptors are coupled with G_i_ which suppresses adenylyl cyclase and reduces cAMP. Activation of presynaptic α_2_-adrenoceptors suppresses the synaptic release of neurotransmitters in various brain regions (Yavich et al., 1997; Nasse and Travers, 2014). α_1_-adrenoceptors activate phospholipase C signaling pathway through G_q_. Dopamine acts on five types of G-protein coupled receptors, D_1_–D_5_, which are categorized in two main functional classes of D_1_ and D_2_ (Beaulieu and Gainetdinov, 2011). D_1_-class of receptors comprises D_1_ and D_5_ which are coupled to G_s_ and enhance cAMP production (D_1_ and D_5_) and phospholipase C activity (D_5_ or D_1_:D_2_ heterodimers, Figure 1C). D_2_-class of dopamine receptors comprises D_2_, D_3_, and D_4_. These receptors are coupled to G_i_ and hence reduce the production of cAMP (Beaulieu and Gainetdinov, 2011). D_1_-class is expressed in the target cells, while D_2_-class is expressed in the presynaptic dopamine terminals as well as in the target cells (Beaulieu and Gainetdinov, 2011). D_1_-class of dopamine receptors share the same stimulating pathways which are used by the β-adrenoceptors (cAMP production) and α_1_-adrenoceptors (G_q_ and PLC, Figure 1C). D_2_-class share the pathways used by α_2_-adrenoceptors which involve inhibitory G-proteins (Beaulieu and Gainetdinov, 2011; Schmidt and Weinshenker, 2014). Dopamine can directly activate α_2_-adrenoceptors in LC and hippocampus (Guiard et al., 2008; El Mansari et al., 2010). Therefore, dissociation of DA and NA functions is particularly hard in areas with high concentrations of both, such as prefrontal cortex and hippocampus.

Dissociation of the physiological effects of DA and NA on target cells may be implemented by mechanisms controlling transmitter overflow and pooling. For example, DA transporter restricts the time course of dopaminergic currents in VTA while decay of noradrenergic currents in LC scales with amplitude due to lower transporter efficacy (Courtney and Ford, 2014). Such temporal variations might be a ground for functional dissociations. However, DA is not always cleared by its specific transporter; In frontal cortex and hippocampus for example, DA is primarily cleared by NA transporters (Morón et al., 2002; Guiard et al., 2008). Notably, both DA and NA can also be cleared by low affinity transporters (Duan and Wang, 2010) which can further shape their actions. Therefore, DA reuptake through shared transporters may cause a similar time course of catecholamine signaling. This can be clarified by comparing the synaptic effects of DA and NA in cortex or hippocampus and identifying transporters involved in these regions.

## DA and NA Innervations and Receptor Expressions Vary Across Brain Area

The pattern of catecholamine receptor expression varies across the brain. For example, noradrenergic projections are sparse in dorsal striatum. Similarly, in the core subregion of nucleus accumbens (NAc), noradrenergic inputs are sparse while considerably dense noradrenergic fibers were found in the shell subregion (Berridge et al., 1997; Nomura et al., 2014). It should be mentioned, however, that the noradrenergic fibers in NAc shell originate mainly from α_2_ group of noradrenergic cells (Delfs et al., 1998). Consistent with the projection patterns, earlier studies showed a low NA concentration in the dorsal regions of striatum, while ventral striatum had a relatively high concentration of NA (Brownstein et al., 1974; Versteeg et al., 1976). The concentration of NA may rise due to a phasic release following novel stimuli, or tonic increase due to the changes in the brain state. Both β_1_ and α_2__c_ type of adrenoceptors are highly expressed in the striatal projection neurons, dopaminergic terminals (Paschalis et al., 2009; Hara et al., 2010) and cholinergic interneurons (Pisani et al., 2003). Such dense receptor expression can make the striatal circuits sensitive to NA signaling despite overall low noradrenergic inputs. We should also consider that DA effects can also be partially mediated by α_2_ adrenoceptors (Cornil et al., 2008; Guiard et al., 2008). Consistently, both DA and NA can modulate the cAMP signaling pathway in the striatum (Nomura et al., 2014). Unlike basal ganglia, corticothalamic regions receive much denser input from LC (Nomura et al., 2014). Cortical regions and layers receive a relatively dense and homogeneous LC projections, while VTA projections are layer and region specific (Nomura et al., 2014). Overall, large overlaps in receptor expression and signaling pathway suggest that DA and NA may mediate similar physiological functions (Table 1). Specificity of their actions might depend on their local concentration, the timing of their release and reuptake and activity of the synaptic terminals.

**TABLE 1:** Similarities in physiological roles of dopamine and noradrenaline.

Physiological role	Dopamine	Noradrenaline
Wakefulness/arousal/brain state	Eban-Rothschild et al., 2016	Aston-Jones and Cohen, 2005; Carter et al., 2010; Fazlali et al., 2016
Attention	Noudoost and Moore, 2011	Berridge and Waterhouse, 2003; Eldar et al., 2013
Memory formation	Vijayraghavan et al., 2007; Shohamy and Adcock, 2010	Berridge and Waterhouse, 2003; Sara, 2009
Memory consolidation	Vijayraghavan et al., 2007; Yamasaki and Takeuchi, 2017	Berridge and Waterhouse, 2003; Sara, 2009
Novelty induced memory encoding	Kempadoo et al., 2016; Takeuchi et al., 2016	Kempadoo et al., 2016; Takeuchi et al., 2016
Reward/addiction	Schultz, 2001, 2007; Berke, 2018; Cox and Witten, 2019	Weinshenker and Schroeder, 2007; Aston-Jones and Kalivas, 2008; Sara and Bouret, 2012; Bouret and Richmond, 2015

## Significance of DA and NA Signaling and Their Functional Similarities

Locus Coeruleus-noradrenaline system is known as the major regulator of wakefulness, vigilance, arousal, and memory formation (Aston-Jones et al., 1999; Berridge and Waterhouse, 2003; Aston-Jones and Cohen, 2005; Sara, 2009; Brown et al., 2012). LC-NA also mediates drug associated memory and reinstatement of the drug seeking behavior in addiction (Weinshenker and Schroeder, 2007; Aston-Jones and Kalivas, 2008). Although VTA-DA system is mainly involved in action and reward processing (Schultz, 2001, 2007; Berke, 2018; Cox and Witten, 2019). It also mediates selective attention, working memory and memory consolidation in cortex and hippocampus (Vijayraghavan et al., 2007; Noudoost and Moore, 2011; Yamasaki and Takeuchi, 2017). These effects may be mediated by parallel catecholamine signaling in multiple target regions.

Convergence of the DA and NA signaling pathways suggest that they might have parallel neurophysiological effects. Indeed, from the very early intracellular studies, complex effects of both NA and DA on neuronal excitability were observed (Madison and Nicoll, 1986; Foote and Morrison, 1987; McCormick and Prince, 1988; McCormick, 1989; Zhou and Hablitz, 1999; Kröner et al., 2005). Depending on the neuronal targets and their specific receptors, DA and NA can modulate various intrinsic currents and hence excitability of the neurons (McCormick, 1989; Cathala and Paupardin-Tritsch, 1999; Seamans and Yang, 2004; Rosenkranz and Johnston, 2006; Arencibia-Albite et al., 2007). The most prominent effect of the NA is the modulation of synaptic transmission and various forms of plasticity (Harley, 1987; Mouradian et al., 1991; Sara, 2009), which are analogous to those of DA (Seamans and Yang, 2004; Tritsch and Sabatini, 2012; Froemke, 2015). In various sensory systems, both dopaminergic and noradrenergic systems were effective in remodeling the tuning properties of the cortical neurons (Bao et al., 2001; Manunta and Edeline, 2004; Edeline et al., 2011; Martins and Froemke, 2015; McBurney-Lin et al., 2019; Waterhouse and Navarra, 2019). Notably, both systems also contribute to the maintenance and transitions of global brain states, wakefulness, arousal, attention, and memory consolidation (Berridge, 2008; Carter et al., 2010; Lee and Dan, 2012; Eban-Rothschild et al., 2016; Fazlali et al., 2016; Sara, 2017; Schicknick et al., 2019). Reward or punishment related stimuli appear to activate catecholamine producing neurons in both LC and VTA (Bouret and Sara, 2004; Sara, 2009; Sara and Bouret, 2012; Bouret and Richmond, 2015). Furthermore, adrenergic and noradrenergic projections to VTA (Mejías-Aponte et al., 2009) may serve to rapidly broadcast behaviorally relevant signals.

In multiple brain regions the activation of adrenergic receptors enhances long term potentiation, working memory, memory consolidation and retrieval (Sara, 2009). These functions are similar to dopaminergic effects on memory processes (Shohamy and Adcock, 2010). It is assumed that the reward related memory consolidation and learning is mediated through VTA-DA system (Schultz, 2001; Berke, 2018). However, LC neuronal activity is also correlated with reward expectation (Bouret and Richmond, 2015). In parallel with VTA-DA system, dopamine release from dense LC terminals in prefrontal cortex and hippocampus may also serve as a strong reward associated signal to facilitate learning and novelty induced memory encoding (Kempadoo et al., 2016; Takeuchi et al., 2016). Overall DA and NA appear to have parallel effects on learning, brain state and reward processing.

## DA and NA Co-Release From the Locus Coeruleus

Locus Coeruleus has long been identified as a noradrenergic center, where majority of the cells produce noradrenaline from dopamine by expressing dopamine-beta-hydroxylase (Figure 1A). A long-standing challenge, however, was to establish whether these cells store and release DA as a co-transmitter together with NA (Figures 1B,C). The first evidence for DA–NA co-release came from measurements of the DA and NA concentrations in the cortical area following psychoactive drugs or LC pharmacological stimulation (Kawahara et al., 2001; Devoto et al., 2005a, 2006). These studies showed that noradrenergic stimulation/suppression causes a parallel change in concentration of both DA and NA in cortical regions. This was often interpreted as a direct or indirect interaction between VTA and LC projections in the cortical area (Kawahara et al., 2001; Chandler et al., 2014; Xing et al., 2016). Such interactions can be due to the direct projections from noradrenergic nuclei to VTA (Mejías-Aponte et al., 2009), control of DA release from dopaminergic terminals via adrenoceptors (Yavich et al., 1997; Ihalainen and Tanila, 2002) and/or competition of NA and DA for the same transporter (Morón et al., 2002; Yamamoto and Novotney, 2002; Borgkvist et al., 2011). Alternatively, it was also hypothesized that DA can be released from LC terminals as a co-transmitter (Devoto et al., 2006).

Earlier pharmacological studies provided strong evidence for DA–NA co-transmission hypothesis; (1) It was shown that the DA levels in parietal, occipital, and cerebellar cortices – which are poorly innervated by DA fibers – were as high as densely innervated medial prefrontal cortex (Devoto et al., 2001). (2) Dopamine receptor antagonists which were known to enhance DA concentration in striatum, were not as effective as adrenergic agonists and antagonists in modifying DA levels in cortex (Kuroki et al., 1999; Devoto et al., 2001, 2003b, 2004). (3) Chemical and electrical alterations of the LC activity significantly modulated the NA and DA concentrations in cortical regions (Kawahara et al., 2001; Devoto et al., 2003a, 2005a,b; Masana et al., 2011). (4) Electrical stimulation of the LC did not change DA concentration in striatum but significantly increased NA level in this region (Devoto et al., 2005a, b). (5) Selective lesion studies and specific disruption of catecholamine production in VTA and LC confirmed that noradrenergic terminals are the main source of dopamine in cortical regions (Pozzi et al., 1994; Devoto et al., 2008, 2015, 2019; Smith and Greene, 2012). Moreover, several medications for neuropsychiatric conditions increase both DA and NA in the brain (Devoto et al., 2006).

Novel genetic and optogenetic techniques make it possible to study the function of neuromodulator nuclei with greater specificity (Carter et al., 2010; Eban-Rothschild et al., 2016). Using these methods, recent studies dissected VTA and LC projections in hippocampus and found that dopamine release from dense LC projections is the primary cause of learning and memory in certain tasks (Kempadoo et al., 2016; Takeuchi et al., 2016; McNamara and Dupret, 2017). Optogenetic stimulation of LC terminals in hippocampus enhanced DA together with NA and specific LC lesion reduced both neurochemicals (Kempadoo et al., 2016). More importantly, these studies for the first time revealed a strong causal link between dopamine release from LC projections and performance in certain learning and memory tasks. Kandle and colleagues showed that photo-stimulation of LC projections in dorsal hippocampus improved mice performance in spatial learning and memory tasks (Kempadoo et al., 2016). At the same time, Morris and colleagues found that LC dopaminergic activity in the hippocampus is necessary for novelty associated memory formation (Takeuchi et al., 2016). Surprisingly, noradrenergic blockade in hippocampus had no effect on either spatial memory or novelty associated memory enhancement (Kempadoo et al., 2016; Takeuchi et al., 2016), despite earlier studies showing beta-adrenergic dependent memory-encoding and plasticity (Straube et al., 2003; Lemon et al., 2009). Overall these studies confirmed the DA–NA co-transmission and the significant role of LC-DA in spatial memory encoding and novelty induced memory consolidation. Whether DA–NA release from LC contributes to distinct memory processes from VTA-DA remains elusive (Yamasaki and Takeuchi, 2017; Duszkiewicz et al., 2019).

## Concluding Remarks and Future Directions

An overview of the studies on dopamine and noradrenaline signaling and function in the CNS suggests that these systems may act in parallel and overlapping manner. Here we provided an integrative approach to support this view. Highly overlapping functions of catecholamines raise important questions; Does LC release DA throughout the brain? What are the functional interactions of LC and VTA? How does the distribution of receptors and transporters of catecholamines determine their specific function? Is ratio of DA/NA important and how can it change? Recent developments of the synthetic and genetically encoded catecholamine sensors (Patriarchi et al., 2018; Sun et al., 2018; Beyene et al., 2019; Feng et al., 2019) together with well-established optogenetic and two-photon imaging techniques make it possible to dissect the role of these neuromodulators in brain computations. Superior spatiotemporal resolution of these sensors makes it possible to examine local/global signaling, volume transmission, co-release and interactions of catecholamines. DA and NA are involved in major brain computations such as sensory processing, motor planning, plasticity, and memory encoding. They are also crucial in mood maintenance, motivation, and concentration.

Future studies should attempt to decode behaviorally relevant signals in LC and VTA and compare the modulatory effects of LC-DA, LC-NA, and VTA-DA in cortex, hippocampus, and ventral striatum. Adrenergic and noradrenergic nuclei other than LC also require a fresh attention in this regard. A better understanding of these systems would facilitate the advent of more effective therapeutics for a range of psychiatric conditions, such as depression (Nutt et al., 2007; Hare et al., 2019), schizophrenia (Winograd-Gurvich et al., 2006; Howes et al., 2017; Matthews et al., 2018), ADHD (Levy, 2009; Tripp and Wickens, 2009), PTSD (Hendrickson and Raskind, 2016), and addiction (Weinshenker and Schroeder, 2007). It is not clear how different neuromodulatory systems are disrupted in these disorders. Research on the overlapping functions of the neuromodulators can provide a new insight into the mechanisms of neuropsychiatric disorders.

## Author Contributions

YR-S and ZF equally contributed to the conception and writing of the manuscript.

## Conflict of Interest

The authors declare that the research was conducted in the absence of any commercial or financial relationships that could be construed as a potential conflict of interest.
